# Building a humane scientific future: Second Asia Laboratory Animal Day

**DOI:** 10.1002/ame2.70134

**Published:** 2026-01-08

**Authors:** Vijay Pal Singh, Himalaya Bhardwaj, R. K. Shakthi Devan, Rahul Thorat

**Affiliations:** ^1^ Laboratory Animal Scientist's Association (LASA) Delhi India; ^2^ Bihar Veterinary College Bihar Animal Sciences University Patna India

**Keywords:** 3R, AFLAS, LASA India

## Abstract

Frontiers in Laboratory Animal Handling and Experimental Design: Bridging Ethics and Excellence

Theme: *Good Science with Good Care*

Ethical Framework:

CCSEA & IAEC oversight | 3Rs (Replacement, Reduction, Refinement) | Transparent documentation

Animal Handling & Anatomy:

Mice • Rats • Rabbits • Guinea pigs

Gentle restraint • Anatomical landmarks

Administration routes—SC, IM, IP, IV (tail vein, ear vein)

Blood sampling—tail vein, saphenous, submandibular/orbital sinus, ear vein/artery

Husbandry & Nutrition:

Balanced diet • Clean water • Environmental enrichment • Hygiene

Health indicators—weight, coat condition, hydration, activity

Experimental Design:

Hypothesis development • Control vs. treatment groups

Randomization • Blinding

Statistical power analysis for ethical sample size

Reproducibility assurance

Pathology & Bio‐Methodology:

Routine health monitoring • Early disease detection

Confounder reduction • Humane techniques

Hands‐On Training:

Handling & restraint practice • Injection simulation

Cage management & enrichment selection

Ethical documentation • Endpoint assessment

OUTCOME

Enhanced animal welfare • Improved data reliability • Ethical compliance •Skilled personnel • Responsible research culture

CORE MESSAGE

GOOD SCIENCE WITH GOOD CARE

*Ethics + Expertise + Experimental Rigor = Responsible Research Excellence*

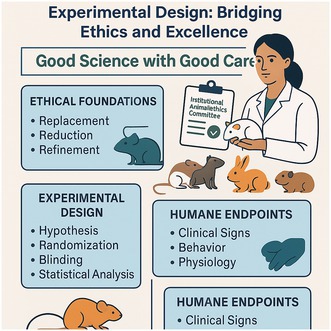

The One‐Day Workshop on “Frontiers in Laboratory Animal Handling and Experimental Design: Bridging Ethics and Excellence” organized on the occasion of the Second Asia Laboratory Animal Day, offered a comprehensive learning experience that seamlessly integrated ethical principles, technical competencies, anatomical knowledge, and scientific methodology into a unified framework for responsible laboratory animal care. Centered around the theme “Good Science with Good Care,” the program highlighted that high‐quality, reproducible research is achievable only when humane practices, strict adherence to regulatory guidelines, and thoughtful experimental planning form the foundation of all laboratory work. The workshop reinforced the idea that scientific excellence and animal welfare are not separate goals, but complementary pillars that together ensure ethical, efficient, and meaningful preclinical research.

The day began with an overview of the ethical foundation that governs laboratory animal use, highlighting national regulatory bodies such as Committee for the Purpose of Control Supervision of Experiment on Animals (CCSEA) and the oversight role of Institutional Animal Ethics Committee (IAEC). Participants were introduced to the essential principles of Replacement, Reduction, and Refinement (3Rs), which encourage the use of alternatives to animal models whenever possible, thoughtful selection of sample size, and improvement of techniques to reduce the discomfort and distress. The importance of accurate completion of various CCSEA forms was emphasized as a core requirement for ensuring accountability, documenting experimental details, and maintaining transparency throughout the research process.

The workshop then progressed into the practical aspects of laboratory animal handling and care. A strong focus was placed on understanding the anatomy and behavioral characteristics of commonly used research species, including mice, rats, rabbits, and guinea pigs. Key anatomical landmarks were discussed not only to ensure safe and effective restraint but also to facilitate accurate administration of substances. Participants reviewed major sites for common experimental procedures, such as subcutaneous areas over the scruff and flank, intramuscular regions like the quadriceps and gluteal muscles, and intraperitoneal injection zones located in the lower abdominal quadrants. Intravenous access points, including the lateral tail vein in rodents and marginal ear vein in rabbits, were also highlighted as essential anatomical regions for both injections and blood sampling.

Blood collection techniques were explained with reference to species‐specific anatomy. Important collection sites included the tail vein and saphenous vein in rodents, the orbital sinus or submandibular region for small‐volume sampling, and the marginal ear vein and central ear artery in rabbits. Emphasis was placed on selecting minimally invasive techniques, using appropriate needle sizes, and adhering to safe blood volume limits to protect animal welfare while ensuring the reliability of collected samples.

The nutritional management of laboratory animals formed another important component of the workshop. Balanced diets, clean water systems, and species‐specific ad libitum feeding strategies were discussed as vital factors influencing health, stress levels, and ultimately the quality of research data. Participants were introduced to indicators of nutritional imbalance, including abnormal weight fluctuations, poor coat condition, hydration status, and activity levels. Proper nutrition, along with environmental enrichment and hygiene, was recognized as a scientific variable that directly affects experimental outcomes and therefore must be monitored with precision.

The discussion then moved toward experimental planning and scientific design. Concepts such as hypothesis formulation, allocation of control and treatment groups, randomization, blinding, and reproducibility were reviewed to highlight how structured methodology improves the reliability and reduces bias. Particular attention was given to the importance of statistical power analysis, which ensures that studies use an appropriate number of animals neither excessive nor insufficient—aligning scientific needs with ethical responsibility.

Participants were also introduced to common pathological conditions encountered in laboratory animals and how such conditions may influence experimental interpretation. Routine health monitoring and early identification of abnormalities were encouraged as essential practices for preventing confounding variables and maintaining humane standards. The final component of this session focused on bio‐methodologies and humane endpoints. Here, the significance of predetermined clinical, behavioral, and physiological criteria was underscored to ensure timely intervention, prevent unnecessary suffering, and uphold ethical standards throughout the duration of an experiment. Strategies for establishing and recognizing humane endpoints were explained, including behavioral changes, weight loss, altered posture, lack of grooming, and physiological deterioration. Clear endpoint criteria help ensure that animal welfare remains central during both the planning and execution of studies.

Throughout the day, hands‐on training played a vital role in reinforcing theoretical concepts. Participants practiced the gentle handling, restraint techniques, anatomical identification, and simulated injection methods using models. They also engaged in cage management tasks, enrichment selection, and the interpretation of welfare indicators. Exposure to real examples of documentation, ethical forms, and endpoint assessment helped bridge the gap between conceptual understanding and practical application.

By the end of the workshop, participants had gained a multidimensional understanding of laboratory animal science that integrated ethics, anatomy, nutrition, handling skills, and experimental design into a unified approach. The knowledge gained can now be implemented in the research planning and execution to improve the humane treatment of animals, enhance the precision and reproducibility of experiments, strengthen documentation practices, and promote a culture of responsibility grounded in scientific and ethical excellence. The training reinforced that **Good Science with Good Care** is not merely a guideline but a continuous commitment that shapes every stage of laboratory animal research—from planning and handling to monitoring, data generation, and final reporting.

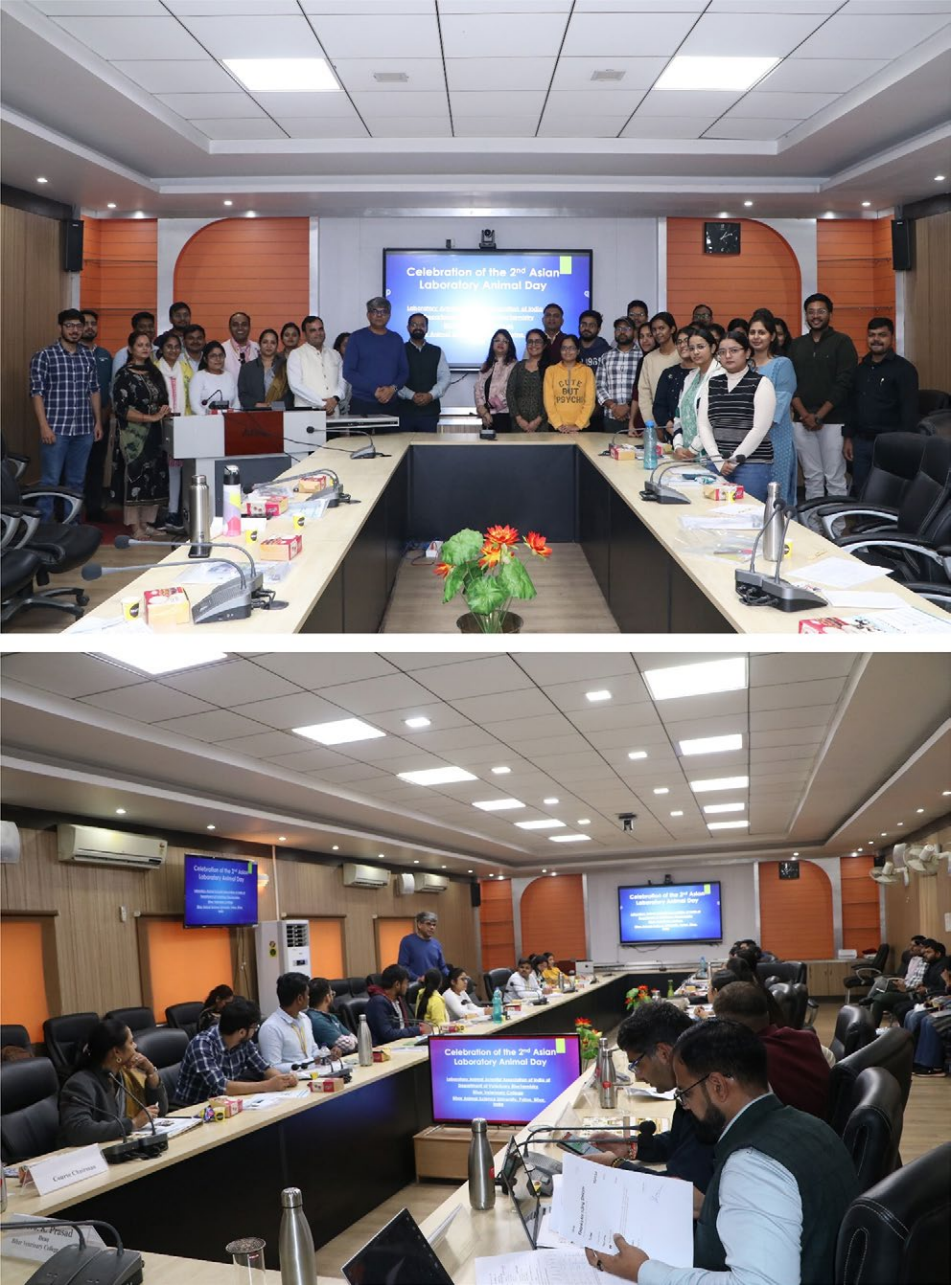

## AUTHOR CONTRIBUTIONS

**Vijay Pal Singh:** Supervision; writing – original draft; writing – review and editing. **Himalaya Bhardwaj:** Methodology; resources; writing – original draft; writing – review and editing. **R. K. Shakthi Devan:** Funding acquisition; methodology. **Rahul Thorat:** Data curation; funding acquisition; resources.

